# Supplementary material for: Dynamics and stage-specificity of between-population gene expression divergence in the Drosophila melanogaster larval fat body
Source: PLoS Genet. 2023 Apr 26;19(4):e1010730. doi: 10.1371/journal.pgen.1010730 (PMC10166500; doi:10.1371/journal.pgen.1010730)
Supplement: S1 Fig — Shown are absolute values of log2 fold-changes of A) genes detected as differentially expressed between the Netherlands (NL, blue) and Zambia (ZI, grey) within early, late and prepupal stages, B) genes with a significant interaction (sig interact) between population and developmental stage versus genes without a significant interaction (no interact) but detected as differentially expressed (DE) between the Netherlands and Zambia during any stage, C) lncRNA and protein-coding (PC) genes detected as differentially expressed between the Netherlands and Zambia within early, late and prepupal stages, and genes detected as privately differentially expressed (private) within the Netherlands or Zambia between D) early and late stages, E) late and prepupal stages, and F) early and prepupal stages. Significance was assessed with a t-test with a Bonferroni multiple test correction. ns not significant, * P < 0.05, ** P < 0.005, *** P < 10−10. (PDF) [file pgen.1010730.s012.pdf]

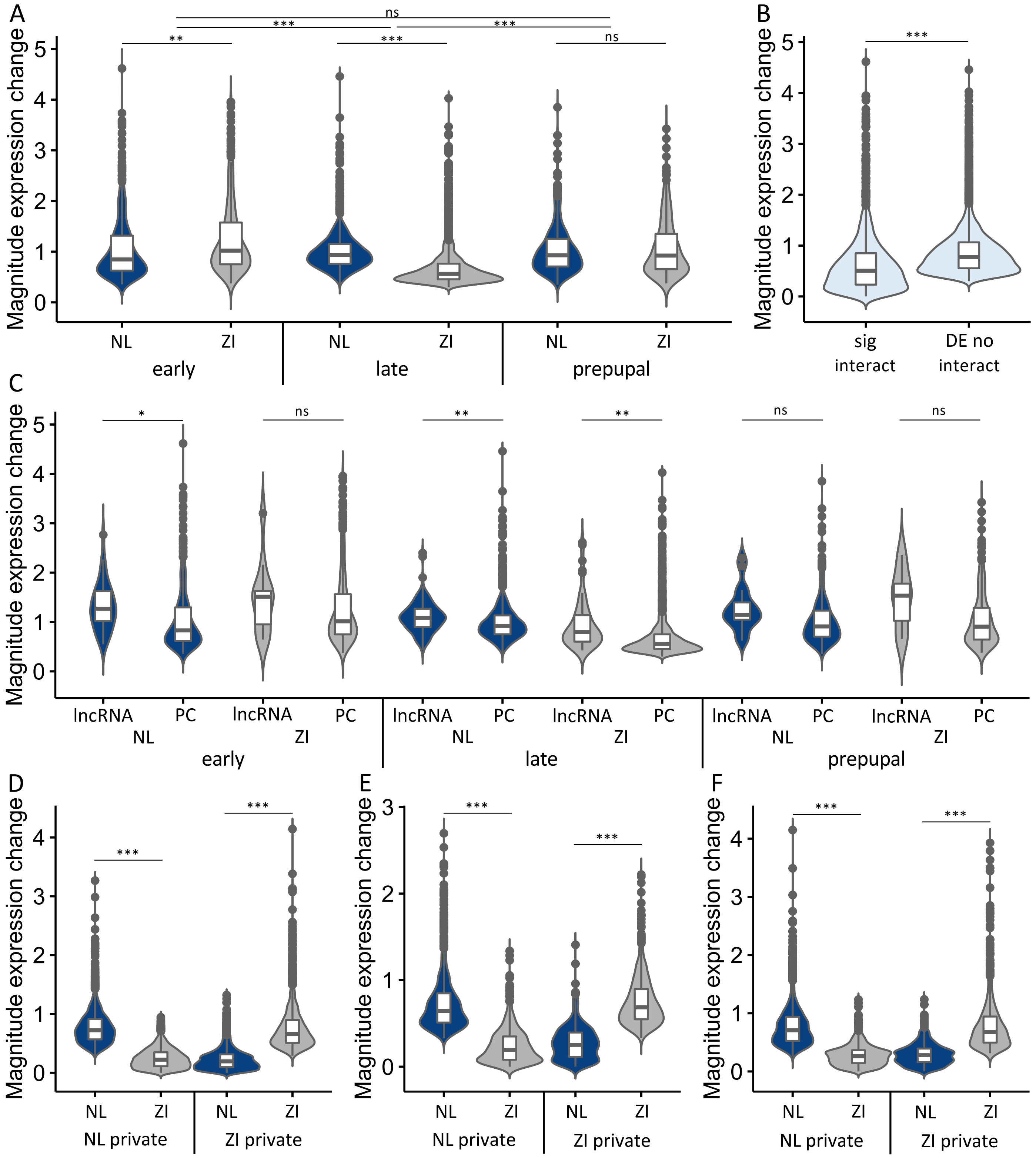

S1 Fig: Magnitude of detected expression changes. Shown are absolute values of log2 fold-changes of A) genes detected as differentially expressed between the Netherlands (NL, blue) and Zambia (ZI, grey) within early, late and prepupal stages, B) genes with a significant interaction (sig interact) between population and developmental stage versus genes without a significant interaction (no interact) but detected as differentially expressed (DE) between the Netherlands and Zambia during any stage, C) lncRNA and protein-coding (PC) genes detected as differentially expressed between the Netherlands and Zambia within early, late and prepupal stages, and genes detected as privately differentially expressed (private) within the Netherlands or Zambia between D) early and late stages, E) late and prepupal stages, and F) early and prepupal stages. Significance was assessed with a *t*-test with a Bonferroni multiple test correction. ns not significant, * $P < 0.05$, ** $P < 0.005$, *** $P < 10^{-10}$.
