# Supplementary material for: Dynamics and stage-specificity of between-population gene expression divergence in the Drosophila melanogaster larval fat body
Source: PLoS Genet. 2023 Apr 26;19(4):e1010730. doi: 10.1371/journal.pgen.1010730 (PMC10166500; doi:10.1371/journal.pgen.1010730)
Supplement: S2 Fig — All analyzed genes were binned in each stage and population combination according to their expression in TPM (very low ≤ 1, low ≤ 10, moderate ≤ 25, high ≤ 50, very high >50). A) All analyzed genes expressed in the Netherlands (NL) or Zambia (ZI) in early, late, and/or pupal stages binned according to their expression level. B) All genes differentially expressed between the Netherlands and Zambia during early, late, or prepupal stages binned according to their expression level. Shown are the number of genes expressed in each stage and population. Blue colors represent genes up-regulated in the Netherlands and grey colors genes up-regulated in Zambia during each respective stage. C) All genes differentially expressed between a given stage and any other stage in either the Dutch or the Zambian population. (PDF) [file pgen.1010730.s013.pdf]

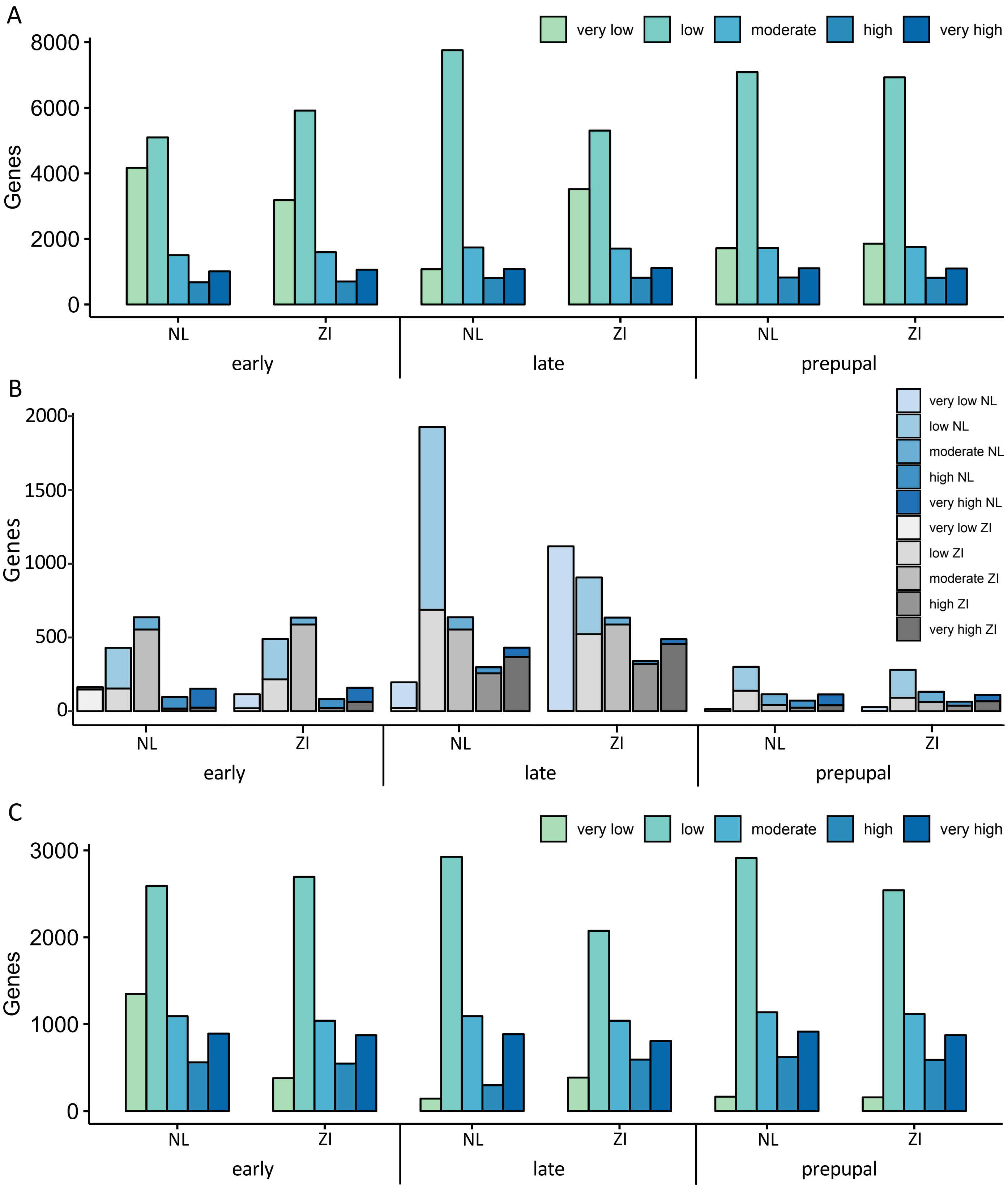

S2 Fig: Distribution of genes across expression bins. All analyzed genes were binned in each stage and population combination according to their expression in TPM (very low ≤ 1, low ≤ 10, moderate ≤ 25, high ≤ 50, very high >50). A) All analyzed genes expressed in the Netherlands (NL) or Zambia (ZI) in early, late, and/or pupal stages binned according to their expression level. B) All genes differentially expressed between the Netherlands and Zambia during early, late, or prepupal stages binned according to their expression level. Shown are the number of genes expressed in each stage and population. Blue colors represent genes up-regulated in the Netherlands and grey colors genes up-regulated in Zambia during each respective stage. C) All genes differentially expressed between a given stage and any other stage in either the Dutch or the Zambian population.
