# Supplementary material for: Dynamics and stage-specificity of between-population gene expression divergence in the Drosophila melanogaster larval fat body
Source: PLoS Genet. 2023 Apr 26;19(4):e1010730. doi: 10.1371/journal.pgen.1010730 (PMC10166500; doi:10.1371/journal.pgen.1010730)
Supplement: S3 Fig — Dutch (blue) and Zambian (yellow) mapping bias for all genes in A) early, B) late, and C) prepupal samples and for only noncoding genes in D) early, E) late, and F) prepupal samples. (PDF) [file pgen.1010730.s014.pdf]

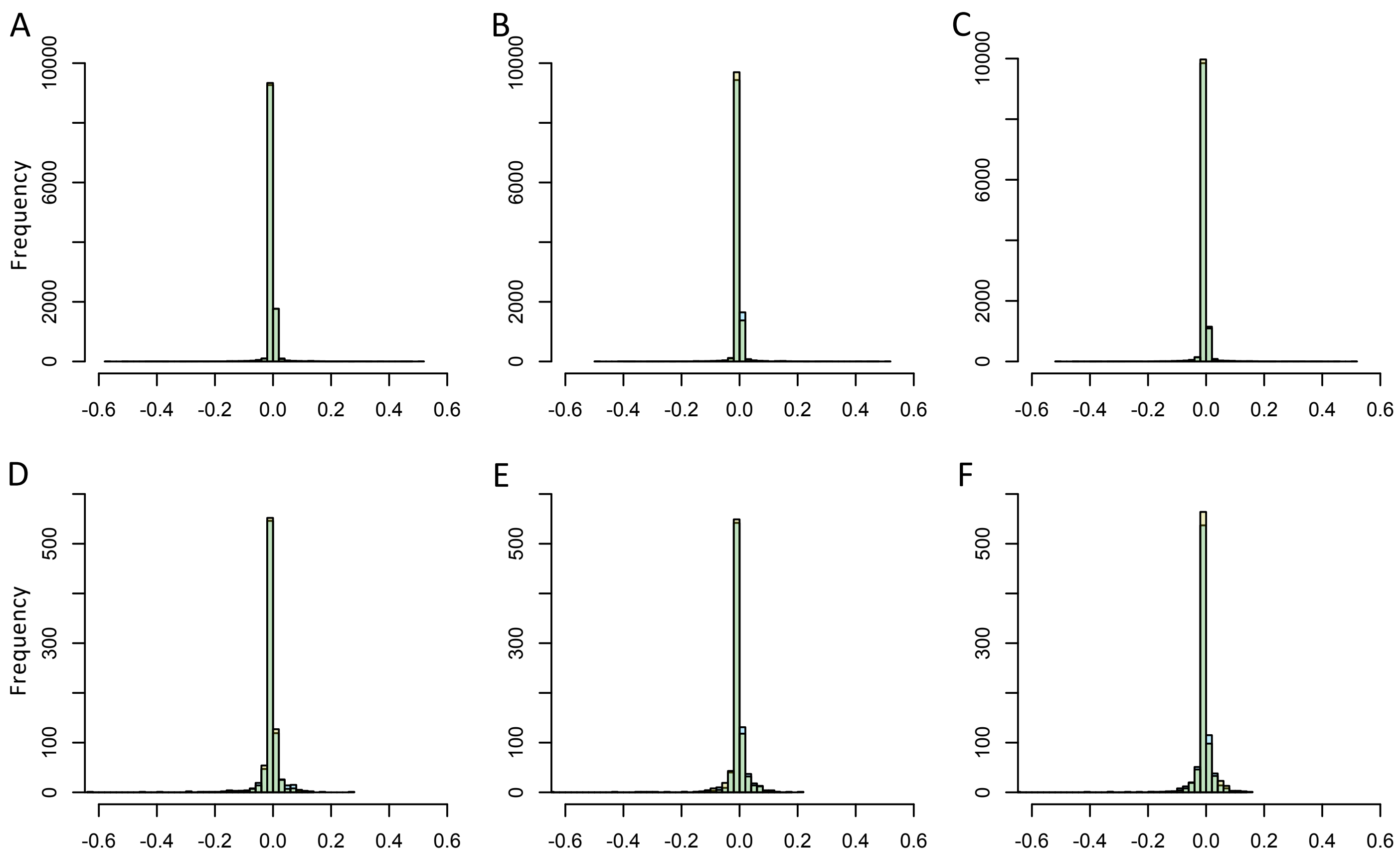

S3 Fig: Distribution of mapping bias. Dutch (blue) and Zambian (yellow) mapping bias for all genes in A) early, B) late, and C) prepupal samples and for only noncoding genes in D) early, E) late, and F) prepupal samples.
