# Supplementary material for: Dynamics and stage-specificity of between-population gene expression divergence in the Drosophila melanogaster larval fat body
Source: PLoS Genet. 2023 Apr 26;19(4):e1010730. doi: 10.1371/journal.pgen.1010730 (PMC10166500; doi:10.1371/journal.pgen.1010730)
Supplement: S4 Fig — A) Hierarchical clustering of module eigengenes. Vertical lines indicate alternative correlation thresholds for module merging 0.85 (red), 0.8 (green), and 0.75 (blue). B) Dendrogram of hierarchical clustering for module assignment. Dynamic Tree cut indicates the colored co-expression modules into which clusters were cut. Using a 0.95 correlation threshold for module merging, the merged modules remained the same as the original cut modules. (PDF) [file pgen.1010730.s015.pdf]

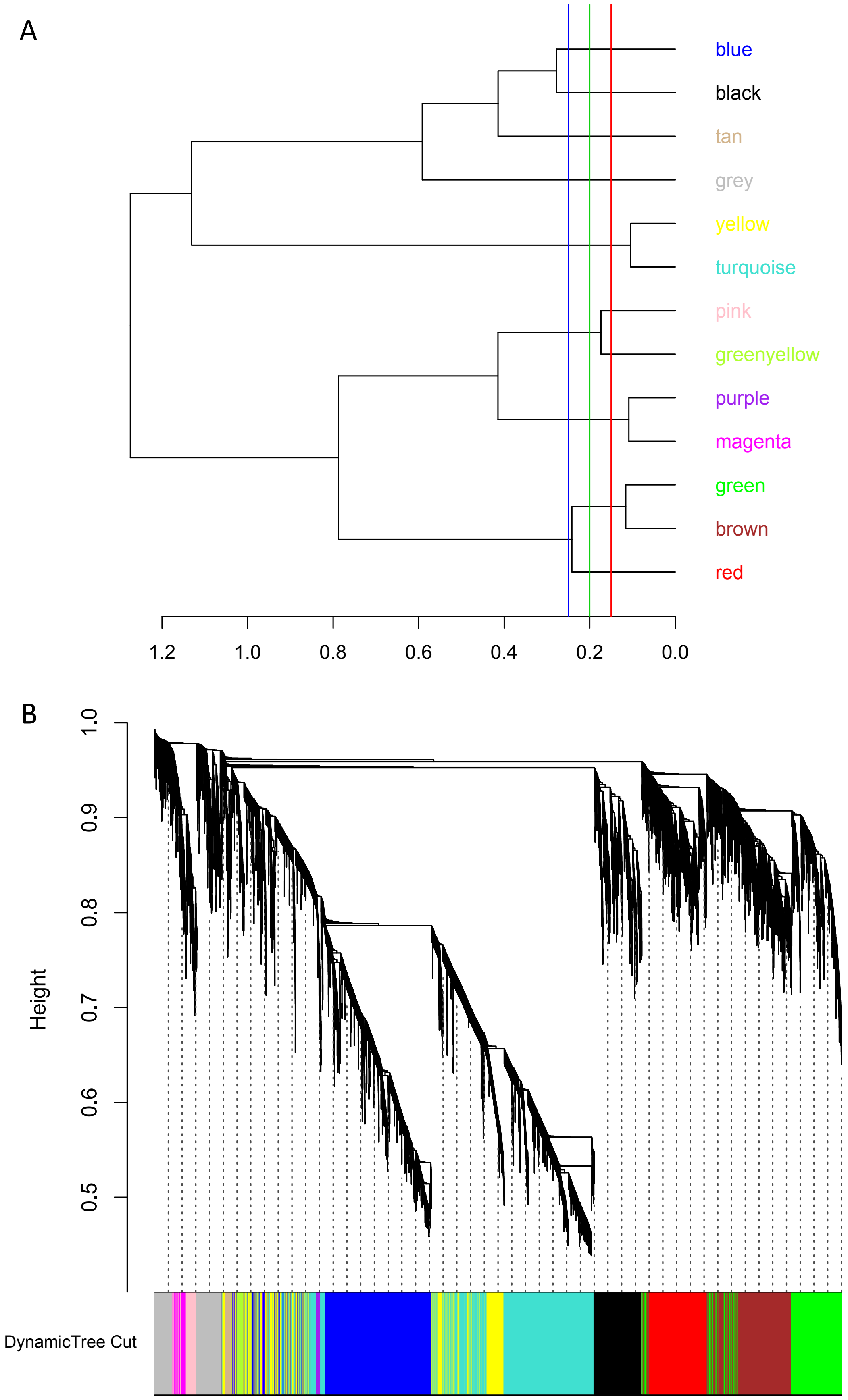

S4 Fig: Gene co-expression modules from WGCNA. A) Hierarchical clustering of module eigengenes. Vertical lines indicate alternative correlation thresholds for module merging 0.85 (red), 0.8 (green), and 0.75 (blue). B) Dendrogram of hierarchical clustering for module assignment. Dynamic Tree cut indicates the colored co-expression modules into which clusters were cut. Using a 0.95 correlation threshold for module merging, the merged modules remained the same as the original cut modules.
