# Supplementary material for: Dynamics and stage-specificity of between-population gene expression divergence in the Drosophila melanogaster larval fat body
Source: PLoS Genet. 2023 Apr 26;19(4):e1010730. doi: 10.1371/journal.pgen.1010730 (PMC10166500; doi:10.1371/journal.pgen.1010730)
Supplement: S1 Text — (PDF) [file pgen.1010730.s016.pdf]

S1 Text. Supplementary Methods and Materials

D. melanogaster samples

For the Dutch and Zambian populations, expression was examined during three developmental stages, representing approximately 10 hours of development: third instar early wandering larvae puff stages 1–2 (L3PS1–2, just after cessation of feeding and onset of wandering), third instar late wandering larvae puff stages 7–9 (L3PS7–9, shortly before onset of pupariation), and the first white prepupal stage (just after puparium formation). The Dutch strains were used in several previous transcriptomic studies [1–4] and the Zambian strains were part of the Drosophila Population Genomics Project [5] and kindly provided by John Pool. Whole genome sequences are available for both populations. Dutch whole genome sequence data are available from http://evol.bio.lmu.de/downloads, while Zambian whole genome sequence data are available from http://www.dpgp.org/. The Munich strains were collected in 2007 and kindly provided by John Baines.

To prevent potential complications from mixed sex samples, only female larvae were used in this study. Larval staging was performed at 25°C. Adult flies were placed in cages and allowed to lay eggs on molasses-agar plates supplemented with yeast for ~48 hours. First instar larvae (identified by their size and mouth hook morphology) were collected and transferred to large vials containing cornmeal-molasses medium supplemented with 5% bromophenol blue (Carl Roth; Karlsruhe, Germany) at a density of 250 larvae per vial. Early and late third instar wandering larvae were identified based on the color of their guts (early: dark blue, late: clear), while early white prepupae (stage P1, before darkening of puparium) were identified by pupariation formation and cessation of wriggling [6].

Divergent lncRNA expression is not due to mapping bias

Because we mapped all of our samples to the FlyBase reference annotation (version 6.21) [7], it is possible that the dearth of up-regulated lncRNAs we detected in Zambia is due to mapping bias from mapping Zambian samples to a lab strain transcriptome; therefore, we remapped our Zambian and Dutch samples to a Zambian reference transcriptome and calculated a simple measure of mapping bias (see Methods section) which yields values between -1 (biased towards Zambian reference) and 1 (biased towards FlyBase reference). We did not detect any significant differences in mapping bias between protein and non-

coding genes within any samples (*t*-test, Bonferroni-adjusted $P > 0.17$ for all). We also did not detect significant differences in mapping bias between the Zambian and Dutch samples when considering either protein-coding or non-coding genes (S3 Fig, *t*-test, Bonferroni-adjusted $P > 0.31$ for all). In both cases, the median mapping bias for all samples was zero, while mean mapping bias was slightly negative for all samples (-0.0005 – -0.0017), regardless of population. For all samples, the mean magnitude of mapping bias was slightly higher for non-coding than for protein-coding genes (0.0167–0.0224 versus 0.0041–0.0067), as was the variance (0.0019–0.0033 versus 0.0005–0.0009), despite the lack of significant differences between coding and non-coding genes within each sample (S1 Data). Thus, the detected dearth of up-regulated lncRNAs in Zambia cannot be explained by differences in mapping bias between Dutch and Zambian samples.

Calculation of developmental stage specificity

Tau was used as a measure of developmental stage specificity. Tau is normally a measure of tissue specificity; however, we have applied it here to measure stage specificity because measures of tissue specificity have previously been successfully applied to measure developmental stage specificity (for examples see: [8, 9]), and a recent study found that out of 9 specificity matrices, tau was the most consistently robust [10].

References

1. Hutter S, Saminadin-Peter SS, Stephan W, Parsch J. Gene expression variation in African and European populations of *Drosophila melanogaster*. Genome Biol. 2008;9(1):R12.

2. Müller L, Hutter S, Stamboliyska R, Saminadin-Peter SS, Stephan W, Parsch J. Population transcriptomics of *Drosophila melanogaster* females. BMC Genomics. 2011;12:81.

3. Catalán A, Hutter S, Parsch J. Population and sex differences in *Drosophila melanogaster* brain gene expression. BMC Genomics. 2012;13:654.

4. Huylmans AK, Parsch J. Population- and sex-biased gene expression in the excretion organs of *Drosophila melanogaster*. G3 (Bethesda). 2014;4(12):2307-15.

5. Pool JE, Corbett-Detig RB, Sugino RP, Stevens KA, Cardeno CM, Crepeau MW, et al. Population Genomics of sub-saharan *Drosophila melanogaster*: African diversity and non-African admixture. PLoS Genet. 2012;8(12):e1003080.

6. Bainbridge SP, Bownes M. Staging the metamorphosis of *Drosophila melanogaster*. Journal of Embryology and Experimental Morphology. 1981;66(1):57.

7. Gramates LS, Marygold SJ, Santos GD, Urbano JM, Antonazzo G, Matthews BB, et al. FlyBase at 25: looking to the future. Nucleic Acids Res. 2017;45(D1):D663-D71.

8. Chen B, Zhang Y, Zhang X, Jia S, Chen S, Kang L. Genome-wide identification and developmental expression profiling of long noncoding RNAs during *Drosophila* metamorphosis. Sci Rep. 2016;6:23330.

9. Svetec N, Cridland JM, Zhao L, Begun DJ. The Adaptive Significance of Natural Genetic Variation in the DNA Damage Response of *Drosophila melanogaster*. PLoS Genet. 2016;12(3):e1005869.

10. Kryuchkova-Mostacci N, Robinson-Rechavi M. A benchmark of gene expression tissue-specificity metrics. Brief Bioinform. 2017;18(2):205-14.
