# Supplementary material for: Dynamics and stage-specificity of between-population gene expression divergence in the Drosophila melanogaster larval fat body
Source: PLoS Genet. 2023 Apr 26;19(4):e1010730. doi: 10.1371/journal.pgen.1010730 (PMC10166500; doi:10.1371/journal.pgen.1010730)
Supplement: Attachment — Submitted filename: Response_to_Reviewers.pdf [file pgen.1010730.s017.pdf]

Dear Dr. Dyer and Dr. Payseur,

We thank you and the reviewers for your/their time and the helpful feedback. Below, please find our response to the reviewers. We have revised our manuscript to address the reviewer comments. Briefly, among other changes, we have added supplementary figures and tables examining the contribution of non-expressed and lowly expressed genes to expression divergence, an analysis of known regulatory regions associated with genes showing strong evidence of population differentiation, and an examination of the magnitude of detected expression changes. Additionally, we have corrected a few typos that we found in our manuscript and added the raw data underlying Fig 5 to our S1 Data file, which was missing from our first submission.

Sincerely,

Amanda Glaser-Schmitt and John Parsch

## Response to Reviewers

**Reviewer #1:** Glaser-Schmitt and Parsch examine gene expression in the larval fat body of two populations of D. melanogaster at three timepoints. The paper is clear and well written and the analyses are well executed. The RNAi experiments are a nice addition to the bioinformatic analyses.

Comments:
Line 556: The inclusion criteria as described does not appear to take into account transcript length, which may bias the inclusion criteria. It looks like this filtering was done prior to the DE analysis. The same issue applies to the calculation of relative gene expression. Also, how often are genes expressed in only one stage or population? How many genes show DE due to lack of expression in one stage or population?

Response 1: The reviewer is correct that we did not take transcript length into account in our inclusion criteria. We designed these criteria in order to retain genes that may be biologically relevant but are expressed in only a subset of the analyzed stages or populations, while at the same time excluding genes with low read counts across all stages or populations in order to minimize the number of multiple statistical comparisons that are performed. Because the statistical analysis performed by DEseq2 relies on count data, filtering genes by read count is a standard approach.

By requiring an average of 15 reads across all replicates, we included genes in our analyses that fell below the 15 read threshold in at least one population and/or stage. In order to determine how much these "non-expressed" genes contribute to the differential expression that we detected, we defined a gene as non-expressed when it had an average of <15 reads in a given stage and population combination and determined how many were also detected as differentially expressed. These results can now be found in S1 Table of the revised manuscript. In any pairwise comparison of the same developmental stage between the two analyzed populations, 42–674 genes (0.33–5.41% of all analyzed genes) were not expressed in one of the populations. 10–186 of these genes were detected as significantly up-regulated in the population in which they were expressed, representing 0.95–11.63% of genes detected as up-regulated in a particular population during a particular stage. Thus, although genes expressed in only one population contribute to genes detected as differentially expressed between the populations, they represent a small minority of the expression divergence that we see. When making pairwise comparisons between two stages within a population, we detected 198–935 (1.59–7.51% of all analyzed genes) as expressed in only one stage, 40–255 (2.67–

6.30%) of which were detected as differentially expressed between the two stages. Please see also response 1 to reviewer 3.

Table 3: Do any of the SNPs identified in the gene regions land in known regulatory elements? Using MEME suite or another program could discover if any of these SNPs are in interesting regions. Additionally you could look for enriched motifs surrounding DE genes.

Response 2: In order to better understand how/if the population differentiation we detected affects known regulatory regions, we focused our analysis on the genes showing the strongest evidence of population differentiation, i.e. those differentially expressed across all examined stages and detected as having an excess of highly differentiated SNPs. First, we identified known, experimentally validated *cis*-regulatory regions associated with these genes using the REDfly database. We identified 78 known regulatory elements, which were associated with 8 (1-47 regulatory regions per gene) of the 35 genes in this group (S8 Table). All but 5 regulatory regions contained highly differentiated SNPs (S8 Table in our revised manuscript). In order to test for an enrichment of regulatory motifs in the identified regions, we use the AME program in the MEME Suite to test for an enrichment of motifs in regulatory regions associated with up-regulated genes in one population versus the other population. We detected an enrichment for 5 motifs, all of which were found in the regulatory regions associated with genes up-regulated in the Zambian population (S9 Table). We have updated our methods and results sections to reflect these new analyses (lines 431–45, 710–7 in our revised manuscript).

Is there any difference in the time the Dutch vs. Zambian strains take to go from the early wandering to prepupal stage? This might bear on the differences in lncRNA expression changes between stages for these two populations especially for the late wandering stage since it is not a fat body developmental milestone.

Response 3: This is a good question. While staging our samples, we detected variation in the timing between the examined stages among isofemale strains within both populations, which is to be expected in natural populations. However, there were no clear differences between populations where one population developed at a faster speed than the other. To control for any variation in timing among isofemale strains, we staged early and late wandering samples using the blue gut method. Briefly, the larvae were raised on medium supplemented with bromophenol blue and were identified based on the color of their guts (early: dark blue, late: clear). Early white prepupae were identified by pupariation formation and cessation of wriggling. Detailed information on how we staged our samples can be found in our Supplementary methods (S1 Supp Text); however, this may have been unclear in our original manuscript. In our revised manuscript we have made it clearer that the detailed sample staging information can be found in the supplementary methods (line 590).

Figure 2B: The MU17 sample seems to be driving a lot of the variance within the German population and is also most different between replicates. Could there be any technical differences between the samples? Does excluding this sample alter your conclusions?

Response 4: The reviewer is correct that in Fig 3B MU17 appears to be the most different from the other isofemale strains as well as have the most variation among biological replicates. However, we should note that it shows similar numbers of differentially expressed genes in comparison to the other isofemale strains (Fig 3A). All of the German samples were collected and sequenced during the same time period and were staged, collected, and sequenced in the same manner. Thus, there should not be any technical differences between any of these samples driving the observed

differences. When we exclude MU17 from the analysis, our conclusions remain the same. When we exclude MU17, we found only very slight differences in the number of differentially expressed genes. Indeed, we still recovered 95–98% of the detected differentially expressed genes for each of the remaining strain comparisons. A summary of these findings can be found in Table R1 below, which we have included as S10 Table in our revised manuscript. Moreover, with MU17 excluded, genes up-regulated in the Netherlands were still significantly less likely to be differentially expressed within Germany than genes up-regulated in Zambia (χ2 test; $P < 10^{-15}$ for all). We have, therefore, left MU17 in these analyses.

| Comparison | | Genes up in strain 1 | | Genes up in strain 2 | |
|---|---|---|---|---|---|
| Stain 1 | Strain 2 | with MU17 | no MU17 | with MU17 | no MU17 |
| MU14 | MU10 | 1029 | 971 | 1035 | 1003 |
| MU14 | MU12 | 1324 | 1296 | 1274 | 1225 |
| MU14 | MU26 | 1110 | 1093 | 1027 | 969 |
| MU12 | MU10 | 1023 | 1006 | 1012 | 959 |
| MU26 | MU10 | 928 | 931 | 813 | 755 |
| MU26 | MU12 | 1250 | 1233 | 1203 | 1167 |

**Table R1: Number of genes detected as differentially expressed in the German population when MU17 is included or excluded in our analysis.** Shown are the number of genes detected as up-regulated in pairwise comparisons of strains (1 and 2) when MU17 is included (with MU17) or excluded (no MU17) in our analysis.

Table 2 - For the genes that show private DE in NL and ZI, do you see a trend in the same direction (LFC) for other population, even if they are non-significant (not DE)?

Response 5: That is a good question. In order to determine if we see a general trend in the same direction in the other population for genes showing private DE between stages in NL or ZI, we performed a Pearson's correlation on the log2 fold-change between stages for genes showing private DE. We found significant correlations between the two populations, suggesting that privately differentially expressed genes tend to show similar patterns in the other population (S7 Table). However, the magnitude of log2 fold-change in these genes was significantly smaller (S1D–F Fig; *t*-test; $P < 2.2 \times 10^{-16}$ for all) in the population in which they were not detected as significantly differentially expressed. Thus, the private changes that we detected between developmental stages within each population likely represent increases in the magnitude of gene expression differences already present in *D. melanogaster*, suggesting that the overall developmental program between these two populations is relatively well conserved. We have presented these new findings in our revised manuscript (lines 385–91, S7 Table, and S1 Figure).

Line 163 - The statement 'shared across at least one stage' is confusing - do you mean shared between at least two stages?

Response 6: Yes. We meant between at least two stages. We have corrected this typo in the revised manuscript (lines 212–3).

Line 290 - "differentially expressed at some time point in at least one population" - I think you mean between two time points in at least one population.

Response 7: Yes. We meant between two time points. We have corrected this in the revised manuscript (lines 366–7).

Figure 4B: can this panel be reordered so that the modules are in the same order as panel A - this would make it easier to quickly compare.

Response 8: Thank you for pointing this out. We have reordered panel B of what is now Fig 2 in our revised manuscript so that the modules are in the same order. Similarly, we have reordered the legend in panel B of what is now Fig 3 in our revised manuscript so that it is in the same order for all panels.

The formatting in tables S1 and S2 needs to be standardized.

Response 9: Presumably the reviewer is referring to several missing borders in what is now Tables S3 and S4 in the previous version of this manuscript. Thank you for pointing this out. We have corrected the formatting in these tables.

**Reviewer #2:** The Glaser-Schmitt and Parsch manuscript investigates gene expression divergence in the Drosophila melanogaster larval fat body across two geographically distinct populations. The evolutionary theory prediction that natural selection is acting more strongly on life stages preceding reproduction provides a strong argument supporting the examination of spatially varying selection at larval stages.
The manuscript feels already well polished, well organized, well written and is easy to read. The methods are sound, the body of data and analysis is significant and going beyond the genomic patterns to the inclusion of single gene validations is a strong plus to the investigation. I am fascinated by the patterns seen regarding to the non-coding genes and the late wandering stages. Given the quality of the manuscript I do not have many comments, however a few major edits could be made, and I am stating below some more comments and lines of thoughts that, I believe, would strengthen the manuscript.

Major points:

Figure 1B is giving me some trouble. I see counts of differentially expressed genes and counts of genes in intersection classes, but I have trouble to grasp where they come from. The text is not helping as the numbers mentioned are not on the figure. Some class comparisons are based on one dot or two or three dots. May be the authors can describe an example in clear words to guide the reader through the figure, or the figure should be edited and made clearer.

Response 1: Thank you for pointing out the potential for reader confusion in this figure. To improve the clarity of Fig 1B, we have added a label beneath the intersection class as well as increased the text size of the label for the significantly differentially expressed genes. We have also added a more thorough explanation for the individual parts of this panel and how they relate to each other, including some examples of how to interpret the intersection class, to the figure legend (lines 156–64).

Line 516-522: Although ribo-depletion is a good approach regarding to studying so-called non-coding genes, I don't think polyA enrichment is redhibitory in studying lncRNAs (the major focus of this paper). Many of the genes annotated as lncRNAs actually do have polyadenylation signals and their

transcripts are captured in regular polyA enrichment protocols. The ribo-depletion protocol however provides access to variation in expression of all the snoRNA, asRNA, miRNA etc that are not polyadenylated but also, as far as I know, poorly documented when regarding to evolution of expression variation along latitudinal gradients.

Response 2: The reviewer is correct that the ribo-depletion approach can be used to detect a broader range of ncRNAs while polyA enrichment can also be used to detect most lncRNAs. Our intention in lines 516–522 was to provide the reader with our justification for using the two different methods for the two experiments, i.e. the focus on both coding and non-coding genes versus the focus on exclusively protein-coding genes. We have reworded this section to make this clearer in the revised manuscript (lines 600–5). The reviewer is also correct that less is known about population differentiation and latitudinal gradients among other types of ncRNAs. However, of the 839 ncRNAs expressed in our dataset, the majority (816) were lncRNAs, leaving only 23 other ncRNAs of various types, with which it would be difficult to perform meaningful analysis. Please see also response 3 to reviewer 2 below.

Line 525: “Reads were mapped to the D. melanogaster transcriptome (including mRNA, rRNAs, microRNAs, and noncoding RNAs)”. I think the authors made all the effort to capture expression variation for non-coding genes. However, the paper is focused only on the lncRNA class. This class is interesting, more and more lncRNAs are characterized and found to encode either encode functional micropeptides, or regulate gene expression for example. But it feels a bit like a missed opportunity that nothing is reported about the other classes of non-coding genes. My suggestion is either the manuscript should state early that the manuscript is only targeted toward lncRNAs for the non-coding part and a short justification for why the authors do so. Or, a better option would be a short paragraph reporting if any pattern is seen (or not) for the different classes of non-coding genes like miRNAs, asRNAs for example that are typically closer to expression regulation.

Response 3: Please also see response 2 to reviewer 2 above. We thank the reviewer for pointing this discrepancy out. Our manuscript focuses on lncRNAs because the majority (~97%) of the ncRNAs that we detected as expressed in our dataset were lncRNAs. However, we failed to mention this important information in our original manuscript. We have now included this information in our revised manuscript in our introduction and results sections (lines 129–30, 294–5). We should also note that for our lncRNA analyses, we included all lncRNAs (including sense and antisense) as defined by FlyBase. Of the 816 lncRNAs in our analysis, 299 were antisense. However, we failed to mention this in our original manuscript. We have made this clearer in our revised manuscript (lines 611–2).

Minor points:
Line 61: There are many papers cited here all under the same topic “all levels of organization”. I think all the papers are relevant here, but a breakdown of the different levels of organization would be giving more perspectives to the reader and clarify what exactly the authors mean.

Response 4: As the reviewer suggests, we have added a breakdown of the various levels of organization to this sentence (lines 61–64).

Given the results highlight strong expression differences between the three larval stages, I think a couple sentences in the introduction reminding the reader about the biological meaning (what the larvae do at each stage) would help strengthening the biological context of this study and fix ideas for the non-specialist readers.

Response 5: Thank you for pointing out this potential issue for readers. We have added a few sentences to our introduction detailing the biological significance of the examined stages (lines 117–123).

Line 447. I feel like the authors are quite (may be overly?) cautious in their discussion. The discussion seems to circle around the idea that non-coding genes by their fast evolutionary pace are good candidates when examining cases of local adaptation over relatively short evolutionary time, but this is not really stated formally. This somehow echoes the abstract sentence "suggesting that lncRNA expression may be more important in derived populations" where "importance" is a little ambiguous. Given how the authors are making the emphasis on the non-coding part of the genome "We examined coding and, for the first time to our knowledge, non-coding gene expression divergence between a population from the species' ancestral range in sub-Saharan Africa and a derived, European population" I could imagine such line of ideas being formally (while still being cautious) presented as hypotheses for example.

Response 6: The reviewer is correct that in our previous manuscript we did not formally state the idea that due to their faster evolutionary rate, non-coding genes can be good candidates for local adaptation. We have now mentioned this idea in our revised discussion (lines 535–6 in our revised manuscript).

Larval fat body cells can contain polytene chromosomes. Could that explain the higher variation in expression seen in the late wandering stage?

Response 7: This is a good question, though difficult to answer. The puffs in polytene chromosomes are known to be regions of high transcriptional activity, with the puffing pattern and transcriptional activity associated with developmental stage. There are also known to be links between replication timing in polytene chromosomes and transcription. As polytene chromosomes are known to be present in all of the examined stages, it is difficult to speculate as to how this might be related to the late wandering stage having higher variation than the other examined stages. One possibility is that there is more variation in replication timing, polytene chromosome number, or puff size in the fat body during the late wandering stage, leading to higher expression variation. However, to our knowledge, there is little information on how replication timing, polytene chromosome number, or puff size vary within developmental stages. Moreover, the detected increased expression variation could also be related to a variety of other mechanisms that influence gene expression variation, such as mechanisms influencing the rate of transcription or RNA degradation. Thus, it is difficult to speculate on the possible underlying mechanisms without more information. In our original manuscript, we failed to mention that the mechanisms underlying increased expression variation specifically during the late wandering stage remain unknown. We have now added this information to our revised manuscript (lines 557–61).

**Reviewer #3:** Genetic changes that create evolutionary phenotypic divergence often act by altering where and when given genes are expressed. Most detailed single-gene and functional genomic studies of this phenomenon have primarily focused on gene expression divergence at macroevolutionary timescales, with a fixed developmental stage being examined. In this paper, Glaser-Schmitt and Parsch investigate how gene expression in the fat body changes across three developmental stages in two populations of Drosophila melanogaster (Zambia, Netherlands). The authors provide empirically supported and interesting insights about gene expression variation across fine-scale developmental and evolutionary time: 1) most gene expression variation found across the samples, as expected, is due to developmental stage rather than population identity, 2) gene expression divergence between populations disproportionately happens during certain

developmental stages, and 3) regulation of long non-coding RNAs (lncRNAs) is as or more likely than proteins to diverge at this evolutionary timescale. The authors lastly demonstrate that at least one of the phenotypes that have diverged between the populations–body size–is affected when some of the differentially expressed genes are perturbed with RNAi.

I found this to be an interesting study that provides important information about how gene expression varies with claims that are supported by the data. The narrow scope of the experiments–one organ, three time stages, and two populations with known demographic histories–strengthens the manuscript by enabling more precise insights and removing other potentially confounding variables. I have some concerns about plausible artifacts that might be affecting the analyses and some suggestions for improving the clarity of the manuscript, articulated below.

Key Points:
The effect of genes at different expression levels on observed patterns. The authors identify large numbers of differentially expressed genes between developmental stages and between populations throughout the paper. The former is to be expected–changing gene expression is a hallmark of development and differentiation. However, the number of differentially expressed genes in both the former and latter case might be artificially inflated by very lowly expressed genes, which tend to be much noisier in RNA-seq datasets. The authors use a cutoff (15 reads minimum per sample for a gene to be considered) but I think an additional plot showing how differentially expressed genes are distributed across expression bins would both show the extent to which this pattern is driven by or robust to changes in lowly expressed genes. For example, the authors write that the vast majority of the 20 most divergently expressed genes between Zambia and Netherlands populations, as measured by relative-fold change, are shared across developmental stages. This is an important result of the paper, but this pattern could be driven by very lowly expressed genes–for such genes, transcript levels may have increased only marginally in magnitude but largely in proportion, or the genes may be prone to being mismeasured because of technical aspects of the experiment. Aside from strengthening the analysis, having this information would provide information about an important feature of gene expression divergence (e.g, do genes at certain expression levels tend to stay conserved while those at others diverge?) and strengthen the paper overall.

Response 1: We thank the reviewer for this suggestion. In order to determine how/if our differential expression analysis results are affected by genes with very low counts, we binned genes according to their expression in TPM (very low ≤ 1, low ≤ 10, moderate ≤ 25, high ≤ 50, very high >50). These results can be seen in S2 Fig. Although lowly expressed genes were present among the genes that we detected as differentially expressed between populations, these genes were not overly represented and did not drive the high number of differentially expressed genes that we detected (S2B Fig). Moreover, these differentially expressed genes with very low expression in one population were usually detected as significantly up-regulated in the other population (i.e. they were not very lowly expressed in the population in which they were significantly up-regulated, S2B Fig). Similarly, when we examined non-expressed genes (i.e. those falling below the 15 read threshold in a population and stage combination), we found that although present, these genes make only a minor contribution to the interpopulation expression divergence that we detected (S1 Table). Please see also response 1 to reviewer 1. Similarly, although lowly expressed and non-expressed genes were present among the genes that we detected as differentially expressed between stages within each population, these genes were not overly represented and did not drive the high number of differentially expressed genes that we detected (S2C Fig). We have updated our results section to reflect these findings (lines 226–31, 367–71 in our revised manuscript). We should also note that our finding that the majority of the top 20 most divergently expressed genes were shared across stages is unlikely to be driven by lowly expressed genes as we detected this pattern regardless of how “most divergent” was defined. We found this pattern for changes detected as the most significant

(i.e. those with the highest *P*-values; S3 Table) as well as those genes with the highest magnitude of fold-changes (S4 Table). Moreover, 10–30% of genes were shared between these two top 20 lists (S3 and S4 Tables). Please see also response 5 to reviewer 3.

Statistical comparisons and multiple testing corrections. The authors perform numerous pairwise comparisons between developmental stages/populations and use a Bonferroni correction (shown in Fig 3). I am unsure of exactly how the authors calculated the statistics for the highlighted differences/non-differences displayed. I think the t-test + Bonferroni correction is accounting for the number of genes being compared between pairwise samples but the authors are also doing a series of t-tests which should be accounted for with its own multiple-testing correction. If the authors have done this correction already, I would urge them to write it with more detail; otherwise this testing burden should be accounted for.

Response 2: We used the Bonferroni multiple-test correction to account for performing multiple *t*-tests; however, this was unclear as written in our previous manuscript. We have made this clearer in our revised manuscript (lines 358–60, 663–4, 671–3; Fig 4 in the revised manuscript).

Effect sizes associated with genetic variants. The authors mention the number of differentially expressed genes that are statistically significant, but the magnitude/effect sizes of the differences are rarely mentioned (the data are presented in the supplementary tables). This information is important for better understanding the types of variants that cause gene-expression divergence and how variants in different developmental stages compare. For example, are there fewer but larger effect size variants between the populations at a certain stage and more but smaller effect size variants at others? Are lncRNAs particularly divergent with respect to magnitude changes? Were the effect-sizes of ~1000 genes with a stage x population interaction (line 301) particularly large? Addressing these questions across the sections the authors see fit is necessary for better understanding the evolutionary history and potential of the regulatory variants and the authors I believe should already have the data from their analyses.

Response 3: We thank the reviewer for the suggestion. We have revised our results section to include the magnitude of the expression differences that we detected between populations (lines 215–26, 313–7, 381–386). These results can be seen in S1 Fig. The magnitude of expression changes between populations was population- and stage-specific (S1 Fig) with stages or populations with higher numbers of differentially expressed genes tending to show lower magnitudes of expression changes in those genes. For instance, the magnitude of expression changes was significantly higher in Zambia during the early stage, but higher in the Netherlands during the late stage (S1A Fig, *t*-test, $P < 0.005$ for both), despite these populations having fewer up-regulated genes than the other population during these stages (Fig 1A). The magnitude of interpopulation expression changes was also significantly higher in the early and prepupal stages than in the late stage (S1A Fig, *t*-test, $P < 10^{-14}$ for both), despite the late stage having a higher number of differentially expressed genes (Fig 1A). When we examined the magnitude of expression changes for genes with an interaction between stage and population, the magnitude of expression changes was significantly smaller than for other genes showing interpopulation differential expression but no interaction with stage (S1B Fig, *t*-test, $P < 10^{-14}$), which underscores that the overall developmental program is well conserved between these two populations. We also confirmed that the magnitude of expression changes between stages in genes privately differentially expressed within one population were significantly higher than the same genes in their counterpart population (S1D–F Fig; *t*-test $P < 10^{-14}$ for all). For lncRNA genes, we found that the magnitude of expression changes in lncRNA genes tended to be larger than for protein coding genes (S1C Fig), which was significant for the Dutch early and late stages as well as the Zambian late stage (*t*-test; $P < 0.05$ for all). Please see also response 5 to reviewer 1.

Other comments:

Line 140: The authors state that "...about 10% could be explained by population." I think they mean 10% of the remaining variation that was unexplained by developmental stage.

Response 4: Thank you for pointing this out. We have corrected this in the text (lines 148–9).

Line 163-164: The authors write "However, when considering the top 20 most divergently expressed genes, as measured by the largest fold changes and/or most significant expression changes."

Response 5: We are unsure what comments the reviewer wished to make about this sentence, but to us the wording of the sentence seems a bit unclear. We have therefore reworded this sentence to make its meaning clearer in our revised manuscript (lines 210–13).

Lines 207-219: The use of the German populations to polarize the changes between Netherhlands and Zambian populations was a really creative experiment and this paragraph greatly strengthens the authors claims. However, some of the sentences are very confusing and I would urge the authors to clarify them and step by the rationale in a more step-by-step way.

Response 6: Thank you. As the reviewer suggests, we have revised this section to make this experiment's rationale and findings clearer (lines 277–84).

Line 313: Further describing tau is important since this might not be a summary statistic that most readers are familiar with. For example, what would the tau value be for genes expressed (weakly or strongly) in 1, 2, or 3 environments?

Response 7: As the reviewer suggests, we have provided some examples of how to interpret tau (lines 408–9).

Lines 327-349: This paragraph largely describes the numbers resulting from the WGCNA analysis without much biological context for making sense of them–I would provide such context whenever possible. A central takeaway from this paragraph is that a samples' developmental stage better explains gene expression variation than population, a result also found in the PCA (and recognized as such lines 348-349). To me, this paragraph should be presented immediately after the PCA. Additionally, I think "age" and "stage" are being used interchangeably in this paragraph, which creates some confusion in the text.

Response 8: As the review suggests, we have moved this paragraph and the accompanying figure forward in the results section so that it immediately follows the results of our PCA. All figures and supplementary tables have been renumbered accordingly. We have also clarified our language in this paragraph to avoid any reader confusion (lines 167–89 in revised manuscript).

Line 407: This paragraph shows important functional data but ends here apruptly. I would urge the authors to summarize the key finding.

Response 9: Thank you for pointing this out. We have now added a short sentence summarizing the key finding of this section to the end of this paragraph (lines 482–3).

Lines 422-427: The language here is a bit imprecise; the detected signatures of potential adaptation (outlier Fst values) are not indicative of changes in cis-regulation between these populations. Rather,

there are regions with higher than expected genetic divergence which are also in the proximity of differentially expressed genes, which implicates divergence in cis-acting regulatory sequences. The Fst values associated with variants say nothing about the variants' mechanism of action; that link is being inferred from other data.

Response 10: The reviewer is correct that it is not the outlier *F*st values themselves that are indicative of *cis*-regulatory changes, but the proximity of these regions with higher than expected genetic divergence to genes that also show population differentiation at the expression level. We have added an additional sentence to make this clearer in our revised manuscript (lines 497–501).

Lines 427: Further, the authors take the increased gene-expression specificity to be adaptive in this section and some of this increase almost certainly is. However, nearly neutral changes are also accumulating between the strains and some of these changes may cause a gene to lose repression during one of the measured stages. Such changes would increase the tau value of a gene, making the gene seem as if it had evolved specificity during adaptation. Because the selection has historically been stronger the in the ancestral, non-bottlenecked population, mutations that are deleterious in Zambia may have evolved as nearly neutral in Netherlands and created such scenarios. This alternative account should be acknowledged.

Response 11: The reviewer is correct that selection in the ancestral Zambian population would have historically been more efficient at removing nearly neutral mutations than in the derived, European population, which experienced a bottleneck as *D. melanogaster* expanded out of Africa. The reviewer is also correct that nearly neutral changes can also alter gene expression, which could increase the value of tau when expression of a gene decreases in a single stage. We should note, however, that the opposite is also true, i.e., a nearly neutral change could also increase expression of a gene in a single stage leading to lower value of tau for that gene. Thus, the proposed scenario would only occur when many nearly neutral changes systematically lead to a large decrease or loss in expression in a single stage, specifically in the Netherlands. Given that we detected similar numbers of non-expressed genes in both populations (see S1 Table), this scenario seems unlikely. That being said, neutral and nearly neutral changes likely do influence our calculation of tau for certain genes and we have now mentioned it as a possibility in our revised discussion section (lines 503–5).

Figure 5: The authors identify genotypes with statistically significant effects from an ANOVA. I think the correct comparison here is actually an ANOVA followed by Tukey's HSD comparing the different genotypes to wildtype. I would recommend this approach because the question is explicitly whether the RNAi genotypes are smaller than wildtype, and this method enables such pairwise comparisons. With this approach, the authors should recover all of the significant differences they have identified but may also detect some of the comparisons from the starvation condition.

Response 12: The reviewer suggests that we compare body size between RNAi knockdown and wildtype flies. We appreciate the idea behind this suggestion; however, body size is known to be a highly polygenic and context-dependent trait. This would make the suggestion difficult to implement and would be unlikely to detect genes affecting body size as significantly different from wildtype unless they have a very large effect size that behaves similarly across different genetic backgrounds. Because body size is so polygenic there is a lot of variation within and among natural populations, making the definition of a single "wildtype" body size difficult to accurately determine. It is also difficult to directly compare the lab strains to wildtype strains since they have such different genetic backgrounds, which will also contribute to variation in body size. Different lab strains and different wildtype strains will have different genetic backgrounds and body sizes, regardless of the expression level of the candidate genes that we examined. Thus, the body size of the knockdown lines cannot

be directly compared to a wildtype strain in a biologically meaningful way. Moreover, due to genetic background-specific effects, the effect of variation in expression of the candidate genes we identify may have different effect sizes within a wildtype background. For RNAi experiments, it is the standard approach to compare the knockdown flies to their siblings who share the same genetic background, aside from whether or not the target RNAi hairpin is expressed. This allows the effect of the target gene knockdown to be determined, while avoiding confounding effects of other variable loci that segregate among natural (and laboratory) strains.

Overall, I think this is an interesting and clearly presented study that describes how genes diverge in expression during the development of an organ between closely related populations and provides empirical information with which to assess models of gene expression evolution.
